# Analysis of Coxsackievirus B5 Infections in the Central Nervous System in Brazil: Insights into Molecular Epidemiology and Genetic Diversity

**DOI:** 10.3390/v14050899

**Published:** 2022-04-26

**Authors:** Raiana S. Machado, Francisco Gomes-Neto, Maria L. Aguiar-Oliveira, Fernanda M. Burlandy, Fernando N. Tavares, Edson E. da Silva, Ivanildo P. Sousa

**Affiliations:** 1 Laboratório de Enterovírus, Instituto Oswaldo Cruz, Fundação Oswaldo Cruz, Rio de Janeiro 21040-900, Brazil; raiana.machado@ioc.fiocruz.br (R.S.M.); fburlandy@ioc.fiocruz.br (F.M.B.); edson@ioc.fiocruz.br (E.E.d.S.); 2 Programa de Pós-Graduação em Medicina Tropical, Instituto Oswaldo Cruz, Fundação Oswaldo Cruz, Rio de Janeiro 21040-900, Brazil; 3 Laboratório de Toxinologia, Instituto Oswaldo Cruz, Fundação Oswaldo Cruz, Rio de Janeiro 21040-900, Brazil; francisco.neto@ioc.fiocruz.br; 4 Laboratório de Vírus Respiratórios e do Sarampo, Instituto Oswaldo Cruz, Fundação Oswaldo Cruz, Rio de Janeiro 21040-900, Brazil; lourdes.oliveira@fiocruz.br; 5 Laboratório de Referência Regional em Enteroviroses, Seção de Virologia, Instituto Evandro Chagas/Serviço de Vigilância Saúde/Ministério de SaúdeS, Ananindeua 67030-000, Brazil; fernandotavares@iec.gov.br

**Keywords:** enterovirus, coxsackievirus B5, central nervous system

## Abstract

Coxsackievirus B5 (CVB5) is one of the most prevalent enteroviruses types in humans and causes annual epidemics worldwide. In the present study, we explored viral genetic diversity, molecular and epidemiological aspects of CVB5 obtained from cerebrospinal fluid and stool samples of patients with aseptic meningitis or acute flaccid paralysis, information that is still scarce in Brazil. From 2005 to 2018, 57 isolates of CVB5 were identified in the scope of the Brazilian Poliomyelitis Surveillance Program. Phylogenetic analyses of VP1 sequences revealed the circulation of two CVB5 genogroups, with genogroup B circulating until 2017, further replaced by genogroup A. Network analysis based on deduced amino acid sequences showed important substitutions in residues known to play critical roles in viral host tropism, cell entry, and viral antigenicity. Amino acid substitutions were investigated by the Protein Variation Effect Analyzer (PROVEAN) tool, which revealed two deleterious substitutions: T130N and T130A. To the best of our knowledge, this is the first report to use in silico approaches to determine the putative impact of amino acid substitutions on the CVB5 capsid structure. This work provides valuable information on CVB5 diversity associated with central nervous system (CNS) infections, highlighting the importance of evaluating the biological impact of certain amino acids substitutions associated with epidemiological and structural analyses.

## 1. Introduction

Most instances of central nervous system (CNS) infections are caused by non-polio enteroviruses (EVs), although other agents can cause neurological illnesses in both children and adults across the world [1]. EVs (genus Enterovirus, family Picornaviridae) are small, non-enveloped, positive sense, and single-stranded RNA viruses. The genomic RNA is around 7.5 kb long in length and encodes a large polyprotein, which is then processed to yield the mature structural (VP1–VP4) and nonstructural proteins. Currently, EVs are categorized into fifteen species (EV A–L and HRV A–C), but only seven can cause human infection [2]. EVs were linked to a variety of clinical manifestations, including hand, foot, and mouth disease (HFMD); acute myalgia; herpangina; conjunctivitis; and severe CNS syndromes such as aseptic meningitis (AM) and acute flaccid paralysis (AFP) [3,4].

Coxsackievirus B5 (CVB5) belongs to the species enterovirus B. The prototype strain (Faulkner) was identified in the USA in a patient with a mild paralytic disease. CVB5, like other EV-species members, is responsible for a wide range of clinical disorders, most of which are self-limiting, ranging from herpangina and hand, foot, and mouth disease to upper and lower respiratory infections [3]. However, it can also cause visceral and neurological disorders such as AFP, AM, viral encephalitis, myopericarditis, and neonatal sepsis-like disease [3,5]. Outbreaks of AM and encephalitis associated with CVB5 have been widely reported worldwide, including fatal cases [6,7,8,9]. Despite its important impact on human health, knowledge of molecular epidemiology and evolution remains very limited, even though significant insights into genetic diversity among CVB5 strains have been discovered in recent years [6,10,11,12,13].

In Brazil, although EVs have long been recognized and characterized as causative agents of CNS infections, there is a limited number of studies on the molecular epidemiology of other EV types besides E30 and E6 [7,8,9]. Furthermore, Brazil has a large territory, and knowledge of the characteristics of the EV types circulating in the country is one of the most important factors in better understanding infection and planning public health policies. In the current study, we assessed the molecular and epidemiological characteristics of CVB5-related CNS infections that occurred in Brazil from 2005 to 2018 within the context of the national EV surveillance system. Our findings give insight into the genetic properties of CVB5 circulating in Brazil, highlighting significant information regarding its genetic characteristics.

## 2. Materials and Methods

### 2.1. Study Background and Virus Isolation

The study comprised clinical and epidemiological information assessed in the scope of the enterovirus surveillance program from the Brazilian Ministry of Health. Cerebrospinal fluid (CSF; *N* = 6.808) and feces (*N* = 8.299) samples were collected from aseptic meningitis and acute flaccid paralysis patients that attended the public hospital network from different Brazilian regions. The samples were sent to the National Reference Laboratory on Polio and non-polio enteroviruses from MoH. Clinical samples were used to inoculate human rhabdomyosarcoma (RD), human cervix carcinoma (Hep2C), and L20B (cell line expressing poliovirus receptor) cell lines as previously described [7,8]. Cell cultures showing cytopathic effect (CPE) were harvested and kept (−20 °C) until typing. EV molecular detection was performed by conventional RT-PCR in the culture supernatant. EV-positive was defined as the presence of CPE followed by conventional reverse transcriptase polymerase chain reaction (RT-PCR) for EVs.

### 2.2. Molecular Detection and Sequencing

Viral RNA was extracted from 140 µL of the culture supernatant using the QIAamp viral RNA mini kit (Qiagen, Hilden, Germany) and used as a template for cDNA construction using random primers (Promega, Madison, WI, USA) and SuperScript III Reverse Transcriptase (Invitrogen, Calrsbad, CA, USA) according to the manufacturer’s instructions. RT-PCR was performed with the primer pairs HEVBS1695 and HEVBR132 (for the entire VP1) [14]. After 40 cycles (94 °C/15 s, 55 °C/20 s, and 72 °C/50 s), PCR products were submitted to electrophoresis, and amplicons were gel-purified (QIAquick Gel Extraction Kit, Qiagen, Hilden, Germany). Cycle sequencing reactions were performed by using the ABI PRISM BigDye Terminator v3.1 Cycle Sequencing Ready Reaction Kit (ABI PRISM BigDye Terminator v.3.1, ABI, Waltham, MA, USA) in a GeneAmp PCR System 9700 thermocycler ((Applied Biosystems, Foster City, CA, USA) with the primer pairs P1S1695S and P2R132S (25 cycles of 96 °C for 10 s, 50 °C for 5 s, and 60 °C for 4 min) [14]. Products were precipitated and examined as previously reported [8].

### 2.3. Phylogenetic Analysis

CVB5 sequences were deposited at the GenBank (NCBI) under the accession numbers OK031005-OK031034 and OK149119-OK149134. Representative CVB5 sequences were downloaded from Genbank (https://www.ncbi.nlm.nih.gov/nucleotide/, accessed on 10 October 2021) and added to our dataset. Faulkner (AF114383.1) was used as reference strain. For phylogenetic reconstruction, sequences were aligned using Muscle [15] available in the Mega 7.0 package [16], and the best nucleotide substitution model was determined by using JModeltest, Version 2.1.4 [17]. The temporal structure of the dataset was verified using TempEst Version 1.5.1 [18]. Afterward, phylogenetic trees were reconstructed by a Bayesian Markov Chain Monte Carlo (MCMC) method, accessible in the BEAST software package, Version 1.10 [19,20]. Time calibration was set based on the year of sample collection, and the general time reversible (GTR) with gamma-distributed rates and invariant sites was employed as the nucleotide substitution model. Beast runs were carried out using the uncorrelated lognormal relaxed molecular clock model and a time-aware Gaussian Markov Random Field (GMRF) Bayesian skyride coalescent tree prior [21,22]. The length of MCMC chains was established as 30 million, sampled every 30,000 steps. Trace files generated through Bayesian phylogenetic inference were visualized and analyzed in Tracer Version 1.7 [23]. Convergence of parameters was considered in the presence of effective sample size (ESS) values exceeding 200. Three independent Beast runs were performed, and .log and .trees output files were further combined (LogCombiner Version 1.10.0). The target Maximum Clade Credibility (MCC) tree was summarized by TreeAnnotator 1.8.4, with a burn-in corresponding to 10% of states. MCC trees were visualized and edited in FigTree, Version 1.4.3 (http://tree.bio.ed.ac.uk/software/figtree/, accessed on 29 October 2021). Beast runs were performed in CIPRES Science Gateway [24]. 

### 2.4. In Silico Analysis

The sequences were aligned by BioEdit (7.2.5.0) and T-Cofee [25,26] and grouped according to amino acid residues. The VP1 sequences were analyzed by changes in chemical characteristics of sidechains—according to the BLOSUM62 substitution matrix [27] in the sidechain volume (A^3^) [28] and hydropathy changes in residues [29]. PROVEAN (Protein Variation Effect Analyzer) approach was used to evaluate the possible structural and functional changes in the VP1 protein compared to the reference sequence [30]. In PROVEAN, a threshold of −2.5 was used (a score ≤ −2.5 was considered deleterious, while a score > −2.5 was considered neutral).

The structure of the asymmetric unit of the prototype strain (Faulkner, AF114383.1) was modeled from the CVB5 F-particle Cryo-EM structure (PDB access 7C9Y) for the Peterborough strain (UniProtKB—Q03053) applying the mutated residue module of VMD—Visual Molecular Dynamics software [31]. The prototypic structure and the consensus sequences were modeled starting from this structure. Images were rendered by VMD and PyMOL (Molecular Graphics System, Version 2.0 Schrödinger, LLC, New York, NY, USA).

## 3. Results

During the last fifteen years (2005–2018), 57 CVB5 isolates (42 CSF and 15 feces) were recovered in clinical samples from patients clinically diagnosed with aseptic meningitis or AFP. Brazilian CVB5 isolates revealed nucleotide and amino acid similarities ranging from 76.3 to 82.4% (mean 77.5%) and above 96.0% in relation to the prototype (Faulkner-AF114383), respectively. CVB5 isolates from 2005 to 2006 showed a lower nucleotide similarity (76.3–78.2%) compared to more recently Brazilian isolates (2017–2018) circulating in the country (81.5–82.4%).

**Figure 1:**
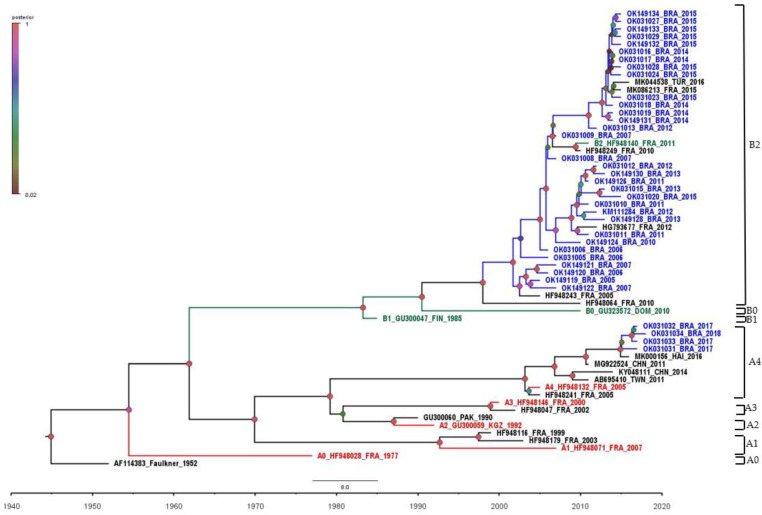
Phylogenetic reconstruction of CVB5 VP1 gene (849 bp), based on Brazilian isolates from meningitis and acute flaccid paralysis cases (represented in blue) and their closely related representative genotypes (represented in red, green and black) and sequences. In the temporal maximum clade credibility (MCC) tree, the posterior probabilities are shown on color and size scale. Scale—the node circle size is proportional to posterior probability support. The strain name, year of sampling, and GenBank accession numbers are also presented.

**Figure 2:**
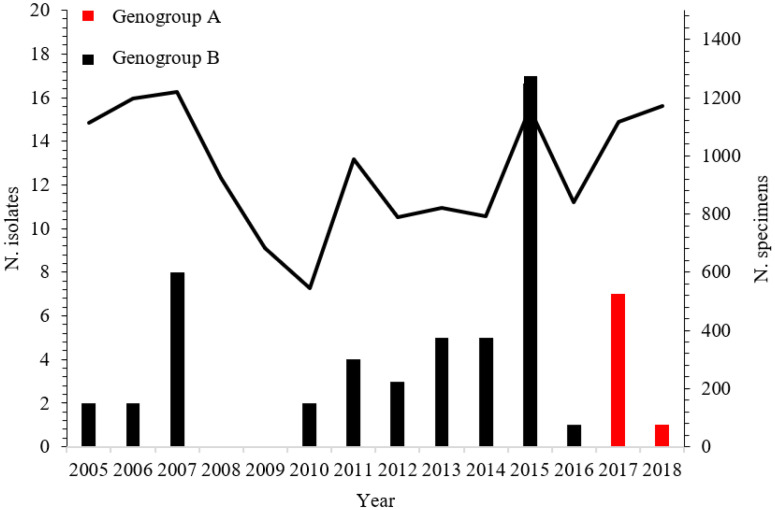
CVB5 distribution among AFP and aseptic meningitis cases in Brazil, 2005–2018. Surveillance data for AFP and aseptic meningitis (black line) and distribution of CVB5 genogroups strains (histogram bars) over time.

The phylogenetic analysis of complete VP1 CVB5 sequences was grouped into two large distinct groups (A and B) and two subgenogroups (A4 and B2) (Figure 1). Genogroup B comprised the majority of sequences assessed in this study and seemed to circulate in Brazil until 2016, being further replaced by genogroup A. The last was composed of four Brazilian CVB5 isolates circulating in 2017–2018, suggesting a more recent introduction of this genogroup (Figure 1 and Figure 2). Additionally, Brazilian CBV5 isolates belonging to subgenogroup A4 were genetically related to viruses previously identified in Haiti in 2016, while subgenogroup B2 grouped with CVB5 identified in France (2010–2012 and 2015) and Turkey (2016) (Figure 1).

**Figure 3:**
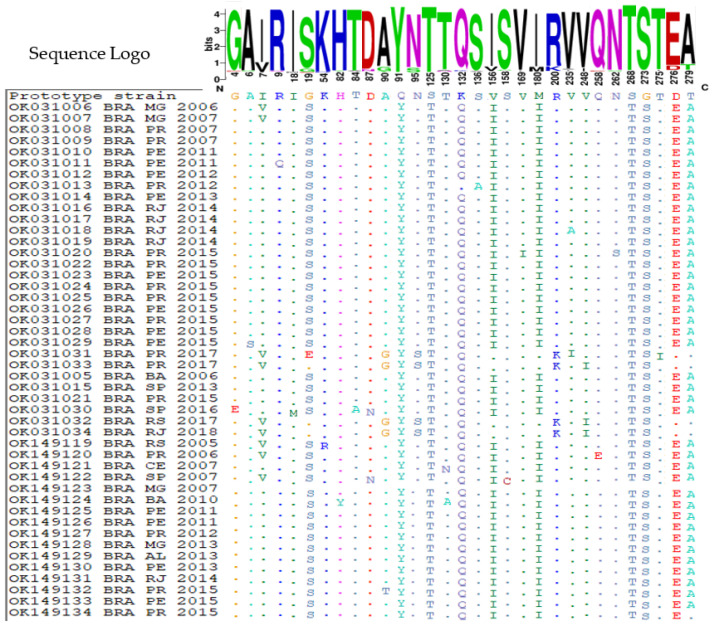
Alignment of deduced amino acid sequences of the VP1 protein from Brazilian CVB5 isolates with the prototype sequence showing variable sites. The dots (.) denote amino acids that are identical to those found in the prototype strain, while the amino acid symbols denote amino acids that are not connected to the prototype. The frequency of aa along the protein is indicated by the letter sequence at the top of the picture.

The amino acid variability in VP1 protein among the Brazilian CVB5 isolates was also explored. VP1 is the most external capsid protein, composed of two important regions: the “canyon” and “pocket factor”—critical structures associated with receptor binding and viral uncoating. Additionally, the VP1 region comprises major neutralizing antigenic sites represented by surface loops—located around the icosahedral five-fold axes, including the BC loop—an important immunogenic site located between the β-sheets B and C [32,33]. Amino acid substitutions within the loop region can confer resistance to neutralization by antibodies directed against the viral capsid [34,35]. Thus, we explored the deduced amino acid sequences from the BC loop and surrounding regions of the prototype strain and our Brazilian CVB5 isolates. The amino acid alignment revealed 251 (89.0%) conserved and 31 (11.0%) variable positions in the VP1 protein compared to the prototype strain (Faulkner strain). In addition, five substitutions were found in the BC loop region (Figure 3). T84A and A90T were only found once, whereas D87N and A90G were observed in two and four CVB5 isolates, respectively. Among CVB5 isolates, the substitution Q91Y was found in all analyzed sequences (Figure 3). As can be shown by the sequence logo program output (Figure 3), parts of amino acids in the prototype sequence were completely replaced by others with similar or different properties.

Based on amino acid sequences, genogroup A of the CVB5 isolates in this study was defined by substitutions I7V, G19E, A90G, Q91Y, N95S, S125T, K132Q, R200Q, V235A, V248A, S268T, G273S, and T275I, whereas the genogroup B was defined by the substitutions G4E, A6S, I7V, R9Q, I18M, G19S, H82Y, T84A, D87N, A90T, A91Y, S125T, T130N/T130A, K132Q, S136A, V156I, S158C, V169I, M180I, V235I, Q258E, N262S, S268T, G273S, D276, and T279A (Table 1). I17V, Q91Y, S125T, K132Q, S268T, and G273S were shared for all Brazilian isolates independently of the genogroup. In addition, different substitutions were observed in the same position: G19E, A90G, V235F (subgenogoup A4) and G19S, A90T, V235A (subgenogoup B2) (Table 1).

Amino acid substitutions at the interaction interface may result in binding affinity changes. For instance, these substitutions can affect the structure of the protein complex, folding protein, antibody virus, and receptor binding. In order to evaluate the putative impact of amino acid substitution on the capsid structure, we carried out in silico analysis with the PROVEAN algorithm. PROVEAN can provide a broad approach to predicting the functional effects of protein sequence variations, such as single or multiple amino acid substitutions [30]. Analysis of the Brazilian sequences identified 35 different amino acid substitutions in the VP1 protein. Of those, only 5.7% (2/35) were predicted by PROVEAN to be structurally or functionally deleterious: T130N and T130A were detected in OK149121 and OK149124 sequences, respectively (Table 1). Conversely, the predicted impact of other substitutions was considered to have a neutral effect, suggesting these variations are not decreasing the viral fitness (Table 1). 

**Figure 4:**
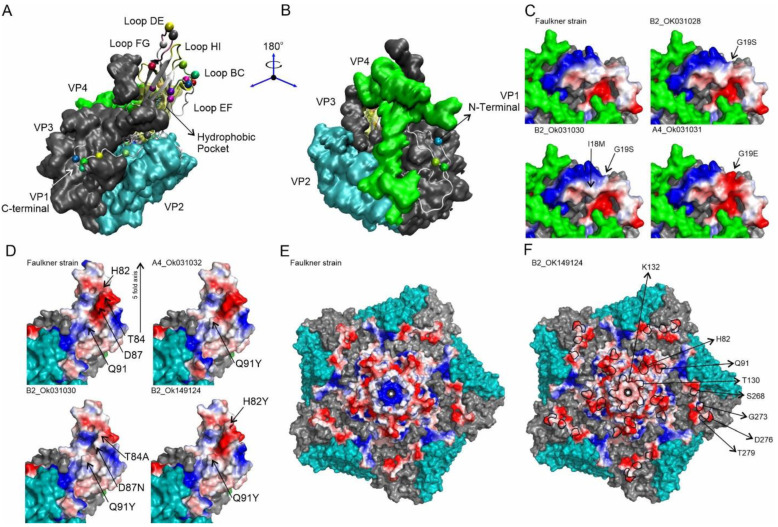
Structural features of VP1 in the asymmetric unit of CVB5 show the sites with modifications and how they are exposed to the virus surface. (**A**) Map of the mutations identified in Brazilian CVB5 isolates on the asymmetric unit of Faulkner prototypic strain with VP1 represented as the tertiary structure and VP2 (cyan), VP3 (gray), and VP4 (green) represented as surfaces. The interacting loops were indicated as BC-Loop (residues from 82 to 90), DE-Loop (residues from 127 to 137), EF-Loop (residues from 148 to 166), FG-Loop (residues from 172 to 177), and HI-Loop (residues from 223 to 230). The position of the hydrophobic pocket, where the palmitic acid molecule is bound, was represented as a yellow transparent surface. Mutations were represented by the residue Cα atom represented as vdW sphere. (**B**) Rotation of 180° showing of the VP1 N-terminus in the capsid inner face. (**C**) VP1 N-terminus was represented as the electrostatic surface showing the Faulkner strain G19 position and representative sequences OK031028, OK031030, and OK31031, showing the substitutions G19S and the sequence I18M, G19S, and G19E. (**D**) BC-Loop was represented as the electrostatic surface showing in detail the position of the mutations and the five-fold axis, the effect of Q91Y mutation represented by OK031032, T84A, D87N, and Q91Y by OK31030 and H82Y by sequence OK149124. (**E**) The five-fold axis for the Faulkner strain, where VP1 was represented as the electrostatic surface, shows the central location of DE-Loop and the positive structural patch (blue) due to the charge of residue K132. (**F**) The five-fold axis for the sequence OK149124 shows the effect of the mutation K132Q, eliminating the central positive patch.

The impact of VP1 protein amino acid changes on the structure of Brazilian CVB5 isolates was also investigated. Figure 4 depicts the substitution positions observed in this study and as they appear in the asymmetric unit on the surface of the virus (Figure 4A). The N-terminus mutations are found on the inner face of the capsid. For instance, G19S was detected in 42 out of 46 sequences and presented a significant change in sidechain volume (48%), including a slight change in electronegativity at this position when compared to the prototypic sequence (Figure 4B,C (B2_OK031028) and Supplementary Table S1). The remaining changes in genogroup B2 were low-frequency mutations, where it is interesting to highlight mutation I18M, which represents a significant change in the hydrophobic nature of this position (Figure 4C (B2_OK031030)). Interestingly, the sequence OK031031 (genogroup A) showed the mutation G19E, which introduced a negative charge at the position and increased sidechain volume significantly (Figure 4C (A4_OK031031) and Supplementary Table S1). 

Regarding BC-loop and adjacent regions, the Brazillian CVB5 isolates presented the substitutions H82Y, T84A, D87N, A90G, A90T, Q91Y, and N95S. Genogroup A4 showed the most impacted sequences, enclosing the cluster of mutations A90G, Q91Y, and N95S. Intriguingly, mutation A90G, which is not on the capsid surface, reduces sidechain volume and hydrophobicity, allowing this loop area to be more flexible (Supplementary Table S1). Simultaneously, these mutations would change the loop flexibility. Mutation N95S, also buried in the structure, causes a loss of charge in this position. The mutation Q91Y exchanges a carboxiamide sidechain to an aromatic ring with a hydroxyl group, increasing the sidechain volume and hydrophobic surface (Figure 4D (A4_OK031032) and Supplementary Table S1) would be interesting for molecular recognition. Low-frequency mutations H82Y, T84A, A90T, and D87N were found in Genogroup B2. In this group, the sequence OK_031030 showed two positions simultaneously mutated (T84A and D87N) (Figure 4D (B2_OK031030)). Figure 4D shows the effect of this mutated patch, which has enhanced hydrophobicity due to T84A and lost negative charge due to D87N (Figure 4D (B2_OK031030). Finally, it is interesting to highlight the mutation H82Y, representing a meaningful change in the hydrophilic character and volume of the position in sequence OK_149124 (Figure 4D (B2_OK149124) and Supplementary Table S1). 

The DE-loop is on the five-fold axis, and it shows two high-frequency substitutions in both genogroups, S125T and K132Q. T130N, T130A, and S136A are three low-frequency mutations found in genogroup B2. The mutation S125T was detected in all reported sequences, showing a slight modification in the hydrophilic character, whereas the mutation K132Q results in a considerable shift in charge distribution along the five-fold axis.

A central positive patch in the prototypic sequence (Figure 4E—Faulkner strain) is substituted by a diffuse negative charged patch (Figure 4F (B2_OK149124)). Two low-frequency mutations in the genogroup B2 that were considered remarkably deleterious by the PROVEAN algorithm showed a significant charge increase (T130N) and a gain in the hydrophobic character (T130A). The relative localization of these mutations was presented in Figure 4F, delimited by the black line, which included the central patch of the K132Q mutation.

The C-terminus is exposed at the virus surface as an interacting region. The high-frequency substitutions S268T and G273S were identified in all 46 sequences. S268T remains the same residue sidechain chemical function with a slight volume change, and G273S increases the hydrophilic character of the position (Figure 4F and Supplementary Table S1). D276E also remains the sidechain chemical function, while the T279A mutation shows a decreased sidechain volume and increased hydrophobicity. The most noticeable changes in Genogroup A4 in opposition to B2 were (i) the absence of mutations in positions D276 and T279, (ii) the mutation R200K, with a decreased sidechain volume, and (iii) a diverging sequence in genogroup A4, OK031031, that includes the low-frequency mutation T275I, showing a dramatic increase in hydrophobicity and sidechain volume change (Supplementary Table S1).

## 4. Discussion

Numerous studies about CNS infection have been conducted in recent years, and non-polio enteroviruses have mostly been reported as an important causative agent associated with sporadic cases and outbreaks [7,8,36,37,38]. In this context, we evaluated the epidemiological and molecular characteristics of CVB5 isolated from CNS infections between 2005 and 2018 in Brazil. 

The phylogenetic analysis of CVB5 Brazilian sequences revealed the circulation of two genogroups of CVB5 in the country. The replacement of genogroups over time was evident in our analyses. Up to 2016, only genogroup B circulated in Brazil. Since the introduction of genogroup A in 2017, however, a virtual switch took place. Our results show that the formation of different genogroups A and B determined in this study by Brazilian isolates was strongly related to the year of isolation. To the best of our knowledge, this is the first report on a change in the circulation pattern of CVB5 in Brazil. 

Due to the high selectivity to escape the immune system, the VP1 gene has low nucleotide similarity among all EV genes [11]. As a result, the complete or partial VP1 encoding region must share >75% nucleotide identity and >85% amino acid similarity to be classified as the same EV type [39,40]. The analysis of our sequences showed a low nucleotide identity (76.3–82.4%). Similar results have already been reported with other EVs, where the nucleotide identity values were relatively low, but the amino acid similarity was quite high [40]. It is worth noting that Brazilian isolates belonging to genogroup A showed a nucleotide similarity higher than genogroup B. Further studies must be conducted to ascertain the putative biological impact of these differences. 

As a result of the low identity verified in the Brazilian isolates compared to the prototype strain, we also analyzed the VP1 protein and the possible substitutions in the different antigenic and receptor binding sites. As previously described, the main changes observed in VP1 are in the N- and C-termini and in loops regions that connect the beta-sheets of the protein’s secondary structure present in conical β-barrel commonly seen in the viral capsid protein [41]. This β-barrel domain is composed of eight antiparallel β-strands (strand B-I), and it plays a critical role in the stability of the virion [5,41,42]. The loops are located on the surface of the virion and are readily accessible to the host immune system [5]. Thus, modifications into loop regions can be associated with escape from neutralization by antibodies and the high antigenic diversity of enteroviruses. Our results showed substitutions in residues 7, 19, 90, 95, 156, 180, 248, 273, 275, 276, and 279. These substitutions were also verified in previous studies [11,39]. In addition, other substitutions were also identified in residues 4, 6, 9, 18, 82, 84, 87, 91, 125, 130, 132, 136, 158, 169, 200, 235, 258, 262, and 268. Some of these substitutions correspond to antigenic sites located within the loop region, and others are in the CAR receptor-binding domain (coxsackievirus-adenovirus receptor). Additionally, substitutions were also observed in the hydrophobic pocket region that may be associated with tropism for neuronal cells [39].

It is worth mentioning that analysis performed through the PROVEAN approach highlighted important substitutions associated with hydropathy, global charge, and volume modifications of the VP1 protein with potential impact on the three-dimensional protein structure. Some were within the loop and βG regions. Additionally, substitutions located on DE-loop were considered deleterious: T130N and T130A. Although these substitutions were considered deleterious, we cannot exclude the possibility of a milder infection or even a loss in viral fitness. These deleterious substitutions in the amino acid residue 130 were found among sequences belonging to genogroup B. The contribution of this event to a loss of viral fitness compared to genogroup A viruses and the putative impact on circulation patterns remains to be established.

Despite the simultaneous change in volume, hydrophobicity, and global protein charge, these changes presented a neutral effect, according to PROVEAN analysis. It is worth noting that even neutral mutations can have an effect when combined with substitutions in other regions of the viral genome. Thus, the biological significance of those substitutions should be further evaluated.

Three major substitutions (Q91Y, S125T, and K132Q) were present in all Brazilian isolates compared to the prototype virus. These substitutions, mainly Q91Y, have already been observed in previous studies with CVB5 and seem to be related to changes in escape/affinity to neutralizing antibodies [43,44]. The substitutions T84A, D87N, A90G, A90T, and Q91Y were specifically located within the BC loop. Previous studies involving EV-A71 revealed that only one substitution (L97R) within the BC loop region was able to increase viral tropism for neuronal cells [45]. Our findings do not allow us to evaluate the possible role of these mutations in escape to antibody neutralization or affinity to neuronal cells, as previously suggested [44,45].

Another remarkable finding was the identification of three substitutions characteristic of Brazilian isolates belonging to genogroup A: A90G, N95S, and R200K. Furthermore, substitutions S268T, G273S, D276E, and T279A are located at the C-terminus of the VP1 protein and exposed on the viral surface. Thus, they can play a critical role in interaction with cell receptors [11]. It is worth noting that the D276E and T279A substitutions were not present in the Brazilian isolates of genogroup A, but their presence in the Brazilian isolates belonging to genogroup B was remarkable. Indeed, some of the substitutions reported, such as A90G (genogroup A) and D276E, T279A, V156I, and M180I (genogroup B), can be the evolutionary pathway of lineages/genogroup [11]. We also identified the substitution R200K as a fingerprint to CVB5 belonging to genogroup A.

It is worth mentioning that although the inferred substitution CVB5 rate is 0.77 × 10^−2^ substitutions per site per year [46], we cannot disregard the possibility the impact of the isolating in cell cultures changes the genome, leading to the identification of sequences that were not due to circulation in human populations.

The difficult access to patient records in order to putatively associate amino acid substitutions with a clinical presentation of the patients, as well as a more thorough patient follow-up to track sequelae emergence in CNS disorders, constituted limiting factors in this investigation.

In conclusion, the present study reveals the circulation pattern of CVB5 in Brazil, as well as the introduction of a new genogroup from 2017 associated with SNC syndromes. We also have demonstrated a higher variation among CVB5 sequences with a potential impact on the three-dimensional protein structure that can be critical for CVB5 neurovirulence and resistance to antibody neutralization. Sequence analyses have led to the identification of a number of viral determinants in enterovirus associated with viral fitness and virulence [47]. Many of these determinants consist of amino acid residues located in the VP1 protein, affecting the early events of virus-host interaction. The putative impact of the substitutions in VP1 sequences, mainly genogroup-specific, is ongoing for a better understanding of virus-host cell interaction. Considering that EV infections are an important public health concern and taking into account their high mutation/recombination rate, EV surveillance policies must improve to timely detect and characterize at the molecular level the circulating of novel emergent viruses.

## Figures and Tables

**Table 1:** Changes and prediction of amino acid substitution impact in the VP1 protein from Brazilian CVB5 isolates.

VP1 Amino Acid Residue		Prototype Strain Residue	Genogroup A					Genogroup B			PROVEAN Prediction
Position	Location		A0	A1	A2	A3	A4	B0	B1	B2	
4	N-terminus	G								G4E	Neutral
6	N-terminus	A								A6S	Neutral
7	N-terminus	I					I7V			I7V	Neutral
9	N-terminus	R								R9Q	Neutral
18	N-terminus	I								I18M	Neutral
19	N-terminus	G					G19E			G19S	Neutral
82	BC-loop	H								H82Y	Neutral
84	BC-loop	T								T84A	Neutral
87	BC-loop	D								D87N	Neutral
90	BC-loop	A					A90G			A90T	Neutral
91	βC-strand	Q					Q91Y			Q91Y	Neutral
95	CD-loop	N					N95S				Neutral
125	βD-strand	S					S125T			S125T	Neutral
130	DE-loop	T								T130A/T130N	Deleterious
132	DE-loop	K					K132Q			K132Q	Neutral
136	DE-loop	S								S136A	Neutral
156	EF-loop	V								V156I	Neutral
158	EF-loop	S								S158C	Neutral
169	βF-strand	V								V169I	Neutral
180	βG-strand	M								M180I	Neutral
200	βH-αF	R					R200K				Neutral
235	βJ-strand	V					V235I			V235A	Neutral
248	C-terminus	V					V248A				Neutral
258	C-terminus	Q								Q258E	Neutral
262	C-terminus	N								N262S	Neutral
268	C-terminus	S					S268T			S268T	Neutral
273	C-terminus	G					G273S			G273S	Neutral
275	C-terminus	T					T275I				Neutral
276	C-terminus	D								D276E	Neutral
279	C-terminus	T								T279A	Neutral

Position: the amino acid residue in the asymmetric unit. Location: the position at the tertiary structure where alfa helices are represented by α and beta strands are represented by β, followed by a sequential alphabetical index. The loop between β-stand and α-helice was represented by βH-αF. Mutations identified inside the secondary structure element were identified by βC-strand, βD-strand, βF-strand, βG strand, and βJ strand. Proto strain residue: the amino acid residue in the prototypic strain. Genogroup: the identified groups in phylogenetic analysis. PROVEAN prediction: the PROVEAN algorithm result for the amino acid residue substitution.

## Data Availability

The data analyzed during the current study are available from the corresponding author on reasonable request.

## Supplementary Materials

The following supporting information can be downloaded at: https://www.mdpi.com/article/10.3390/v14050899/s1, Table S1. In silico Analysis of Virus Asymmetric Unit Mutations [29].

## Abbreviations

A6S	Replacement of alanine by serine at position 6 of the VP1 protein
A90G	Replacement of alanine by glycine at position 90 of the VP1 protein
A90T	Replacement of alanine by threonine at position 90 of the VP1 protein
D87N	Replacement of aspartic acid by asparagine at position 87 of the VP1 protein
D276E	Replacement of aspartic acid by glutamic acid at position 276 of the VP1 protein
G4E	Replacement of glycine by glutamic acid at position 4 of the VP1 protein
G19E	Replacement of glycine by glutamic acid at position 19 of the VP1 protein
G19S	Replacement of glycine by serine at position 19 of the VP1 protein
G273S	Replacement of glycine by serine at position 273 of the VP1 protein
H82Y	Replacement of histidine by tyrosine at position 82 of the VP1 protein
I7V	Replacement of isoleucine by valine at position 7 of the VP1 protein
I18M	Replacement of isoleucine by methionine at position 18 of the VP1 protein
K132Q	Replacement of lysine by glutamine at position 132 of the VP1 protein
M180I	Replacement of methionine by isoleucine at position 180 of the VP1 protein
N95S	Replacement of asparagine by serine at position 95 of the VP1 protein
N262S	Replacement of asparagine by serine at position 262 of the VP1 protein
Q91Y	Replacement of glutamine by tyrosine at position 91 of the VP1 protein
Q258E	Replacement of glutamine by glutamic acid at position 258 of the VP1 protein
R9Q	Replacement of arginine by glutamine at position 9 of the VP1 protein
R200K	Replacement of arginine by lysine at position 200 of the VP1 protein
S125T	Replacement of serine by threonine at position 125 of the VP1 protein
S136A	Replacement of serine by alanine at position 136 of the VP1 protein
S268T	Replacement of serine by threonine at position 268 of the VP1 protein
T84A	Replacement of threonine by alanine at position 84 of the VP1 protein
T130A	Replacement of threonine by alanine at position 130 of the VP1 protein
T130N	Replacement of threonine by asparagine at position 130 of the VP1 protein
T275I	Replacement of threonine by isoleucine at position 275 of the VP1 protein
T279A	Replacement of threonine by alanine at position 279 of the VP1 protein
V156I	Replacement of valine by isoleucine at position 156 of the VP1 protein
V235I	Replacement of valine by isoleucine at position 235 of the VP1 protein
V235A	Replacement of valine by alanine at position 235 of the VP1 protein
V248A	Replacement of valine by alanine at position 248 of the VP1 protein

## References

[1] Koyuncu O.O., Hogue I.B., Enquist L.W. (2013). Virus infections in the nervous system. Cell Host Microbe.

[2] ICTV. https://talk.ictvonline.org/.

[3] Tapparel C., Siegrist F., Petty T.J., Kaiser L. (2013). Picornavirus and enterovirus diversity with associated human diseases. Infect. Genet. Evol..

[4] Sousa I.P., Maximo A.C.B., Figueiredo M.A.A., Maia Z., da Silva E.E. (2019). Echovirus 30 detection in an outbreak of acute myalgia and rhabdomyolysis, Brazil 2016–2017. Clin. Microbiol. Infect..

[5] Pallansch M.A., Roos R.P., Knipe D.M., Howley P.M. (2001). Enteroviruses: Polioviruses, Coxsackieviruses, Echoviruses, and Newer Enteroviruses. Fields Virology.

[6] Liu N., Jia L., Yin J., Wu Z., Wang Z., Li P., Hao R., Wang L., Wang Y., Qiu S. (2014). An outbreak of aseptic meningitis caused by a distinct lineage of coxsackievirus B5 in China. Int. J. Infect. Dis..

[7] Ramalho E., Sousa I., Burlandy F., Costa E., Dias A., Serrano R., Oliveira M., Lopes R., Debur M., Burger M. (2019). Identification and Phylogenetic Characterization of Human Enteroviruses Isolated from Cases of Aseptic Meningitis in Brazil, 2013–2017. Viruses.

[8] Sousa I.P., Oliveira M.L.A., Burlandy F.M., Machado R.S., Oliveira S.S., Tavares F.N., Gomes-Neto F., da Costa E.V., da Silva E.E. (2020). Molecular characterization and epidemiological aspects of non-polio enteroviruses isolated from acute flaccid paralysis in Brazil: A historical series (2005–2017). Emerg. Microbes Infect..

[9] Dos Santos G.P., da Costa E.V., Tavares F.N., da Costa L.J., da Silva E.E. (2011). Genetic diversity of Echovirus 30 involved in aseptic meningitis cases in Brazil (1998–2008). J. Med. Virol..

[10] Rezig D., Fares W., Seghier M., Yahia A.B., Touzi H., Triki H. (2011). Update on molecular characterization of coxsackievirus B5 strains. J. Med. Virol..

[11] Henquell C., Mirand A., Richter J., Schuffenecker I., Böttiger B., Diedrich S., Terletskaia-Ladwig E., Christodoulou C., Peigue-Lafeuille H., Bailly J.L. (2013). Phylogenetic patterns of human coxsackievirus B5 arise from population dynamics between two genogroups and reveal evolutionary factors of molecular adaptation and transmission. J. Virol..

[12] Ma H., Huang X., Kang K., Li X., Tang X., Ren Y., Wang Y., Zhao G., Xu B. (2013). Recombination in human coxsackievirus B5 strains that caused an outbreak of viral encephalitis in Henan, China. Arch. Virol..

[13] Shen H. (2018). Recombination analysis of coxsackievirus B5 genogroup C. Arch. Virol..

[14] Mirand A., Archimbaud C., Henquell C., Michel Y., Chambon M., Peigue-Lafeuille H., Bailly J. (2006). Prospective identification of HEV-B enteroviruses during the 2005 outbreak. J. Med. Virol..

[15] Edgar R.C. (2004). MUSCLE: Multiple sequence alignment with high accuracy and high throughput. Nucleic Acids Res..

[16] Kumar S., Stecher G., Tamura K. (2016). MEGA 7: Molecular evolutionary genetics analysis version 7.0 for bigger datasets. Mol. Biol. Evol..

[17] Darriba D., Taboada G., Doallo R., Posada D. (2012). jModelTest 2: More models, new heuristics and parallel computing. Nat. Methods.

[18] Rambaut A., Lam T.T., Carvalho L.M., Pybus O.G. (2016). Exploring the temporal structure of heterochronous sequences using TempEst (formerly Path-O-Gen). Virus Evol..

[19] Drummond A.J., Rambaut A. (2007). BEAST: Bayesian evolutionary analysis by sampling trees. BMC Evol. Biol..

[20] Suchard M., Lemey P., Baele G., Ayre D.L., Drummond A.J., Rambaut A. (2018). Bayesian phylogenetic and phylodynamic data integration using BEAST 1.10. Virus Evol..

[21] Drummond A.J., Nicholls G.K., Rodrigo A.G., Solomon W. (2002). Estimating mutation parameters population history and genealogy simultaneously from temporally spaced sequence data. Genetics.

[22] Minin V.N., Bloomquist E.W., Suchard M.A. (2008). Smooth skyride through a rough skyline: Bayesian coalescent-based inference of population dynamics. Mol. Biol. Evol..

[23] Rambaut A., Drummond A.J., Xie D., Baele G., Suchard M.A. (2018). Posterior summarisation in Bayesian phylogenetics using Tracer 1.7. Syst. Biol..

[24] Miller M.A., Pfeiffer W., Schwartz T. Creating the CIPRES Science Gateway for inference of large phylogenetic trees. Proceedings of the Gateway Computing Environments Workshop (GCE).

[25] Hall T.A. (1999). BIOEDIT: A user-friendly biological sequence alignment editor and analysis program for Windows 95/98/NT. Nucleic Acids Symp. Ser..

[26] Notredame C., Higgins D.G., Heringa J. (2000). T-Coffee: A novel method for fast and accurate multiple sequence alignment. J. Mol. Biol..

[27] Henikoff S., Henikoff J.G. (1992). Amino acid substitution matrices from protein blocks. Proc. Natl. Acad. Sci. USA.

[28] Zamyatnin A.A. (1972). Protein volume in solution. Prog. Biophys. Mol. Biol..

[29] Kyte J., Doolittle R.F. (1982). A simple method for displaying the hydropathic character of a protein. J. Mol. Biol..

[30] Choi Y., Sims G.E., Murphy S., Miller J.R., Chan A.P. (2012). Predicting the functional effect of amino acid substitutions and indels. PLoS ONE.

[31] Humphrey W., Dalke A., Schulten K. (1996). VMD: Visual molecular dynamics. J. Mol. Graph..

[32] Yuan J., Shen L., Wu J., Zou X., Gu J., Chen J., Mao L. (2018). Enterovirus A71 Proteins: Structure and Function. Front. Microbiol..

[33] Jiang P., Liu Y., Ma H.C., Paul A.V., Wimmer E. (2014). Picornavirus morphogenesis. Microbiol. Mol. Biol. Rev..

[34] Minor P.D., Schild G.C., Bootman J., Evans D.M., Ferguson M., Reeve P., Spitz M., Stanway G., Cann A.J., Hauptmann R. (1983). Location and primary structure of a major antigenic site for poliovirus neutralization. Nature.

[35] Minor P.D., Ferguson M., Evans D.M., Almond J.W., Icenogle J.P. (1986). Antigenic structure of polioviruses of serotypes 1, 2 and 3. J. Gen. Virol..

[36] Fernandez-Garcia M.D., Kebe O., Fall A.D., Ndiaye K. (2017). Identification and molecular characterization of non-polio enteroviruses from children with acute flaccid paralysis in West Africa, 2013–2014. Sci. Rep..

[37] Kapoor A., Victoria J., Simmonds P., Slikas E., Chieochansin T., Naeem A., Shaukat S., Sharif S., Alam M.M., Angez M. (2008). A highly prevalent and genetically diversified Picornaviridae genus in South Asian children. Proc. Natl. Acad. Sci. USA.

[38] Sousa I.P., Dos Santos F.B., de Paula V.S., Vieira T.C.R.G., Dias H.G., Barros C.A., da Silva E.E. (2021). Viral and Prion Infections Associated with Central Nervous System Syndromes in Brazil. Viruses.

[39] Huang H.W., Chu P.H., Pan C.H., Wang C.F., Lin C.C., Lu P.L., Chen Y.S., Shi Y.Y., Su H.J., Chou L.C. (2018). Evolutionary histories of coxsackievirus B5 and swine vesicular disease virus reconstructed by phylodynamic and sequence variation analyses. Sci. Rep..

[40] Angez M., Shaukat S., Zahra R., Khurshid A., Sharif S., Alam M.M., Zaidi S.S. (2015). Molecular epidemiology of enterovirus B77 isolated from non polio acute flaccid paralytic patients in Pakistan during 2013. Infect. Genet. Evol..

[41] Oberste M.S. (2008). Comparative genomics of the coxsackie B viruses and related enteroviruses. Curr. Top. Microbiol. Immunol..

[42] Cifuente J.O., Moratorio G. (2019). Evolutionary and Structural Overview of Human Picornavirus Capsid Antibody Evasion. Front. Cell Infect. Microbiol..

[43] Battistone A., Buttinelli G., Fiore S., Amato C., Bonomo P., Patti A.M., Vulcano A., Barbi M., Binda S., Pellegrinelli L. (2014). Sporadic isolation of sabin-like polioviruses and high-level detection of non-polio enteroviruses during sewage surveillance in seven Italian cities, after several years of inactivated poliovirus vaccination. Appl. Environ. Microbiol..

[44] Norder H., Bjerregaard L., Magnius L., Lina B., Aymard M., Chomel J.J. (2003). Sequencing of ’untypable’ enteroviruses reveals two new types, EV-77 and EV-78, within human enterovirus type B and substitutions in the BC loop of the VP1 protein for known types. J. Gen. Virol..

[45] Cordey S., Petty T.J., Schibler M., Martinez Y., Gerlach D., van Belle S., Turin L., Zdobnov E., Kaiser L., Tapparel C. (2012). Identification of site-specific adaptations conferring increased neural cell tropism during human enterovirus 71 infection. PLoS Pathog..

[46] Lukashev A.N., Vakulenko Y.A. (2017). Molecular evolution of types in non-polio enteroviruses. J. Gen. Virol..

[47] Ang P.Y., Chong C.W.H., Alonso S. (2021). Viral determinants that drive Enterovirus-A71 fitness and virulence. Emerg. Microbes Infect..

