# Supplementary material for: Analysis of Coxsackievirus B5 Infections in the Central Nervous System in Brazil: Insights into Molecular Epidemiology and Genetic Diversity
Source: Viruses. 2022 Apr 26;14(5):899. doi: 10.3390/v14050899 (PMC9146130; doi:10.3390/v14050899)
Supplement: Supplementary file 1 [file viruses-14-00899-s001.zip › viruses-1665442-Supplementary.pdf]

viruses

MDPI

Supplementary Materials

Analysis of Coxsackievirus B5 Infections in the Central Nervous System in Brazil: Insights into Molecular Epidemiology and Genetic Diversity

Table S1. In silico Analysis of Virus Asymmetric Unit Mutations.

MUT	%(VOL)	1-hydropathy	2-hydropathy	Provean Result	Faulkner SASA%	Mutation SASA%
**G4E**	130.28	−0.4	−3.50	Neutral	NC	NC
**A6S**	0.45	1.8	−0.8	Neutral	NC	NC
**I7V**	−16.02	4.5	4.2	Neutral	NC	NC
**R9Q**	−17.07	−4.5	−3.5	Neutral	NC	NC
**I18M**	−2.28	4.5	1.9	Neutral	62.05	79.11
**G19E**	130.28	−0.4	−3.50	Neutral	53.36	63.01
**G19S**	48.09	−0.4	−0.8	Neutral	53.36	49.00
**K54R**	2.85	−3.9	−4.5	Neutral	36.45	56.61
**H82Y**	26.37	−3.2	−1.3	Neutral	51.26	45.98
**T84A**	−23.69	−0.7	1.8	Neutral	71.24	65.76
**D87N**	2.70	−3.50	−3.5	Neutral	55.96	58.80
**A90G**	−32.17	1.8	−0.4	Neutral	4.79	3.81
**A90T**	31.04	1.8	−0.7	Neutral	4.79	3.81
**Q91Y**	34.63	−3.5	−1.3	Neutral	44.26	54.42
**N95S**	−22.00	−3.5	−0.8	Neutral	6.77	8.01
**S125T**	30.45	−0.8	−0.7	Neutral	3.56	4.79
**T130A**	−23.69	−0.7	1.8	Deleterious	81.04	88.36
**T130N**	−1.72	−0.7	−3.5	Deleterious	81.04	75.59
**K132Q**	−14.71	−3.9	−3.5	Neutral	53.91	47.17
**S136A**	−0.45	−0.8	1.8	Neutral	21.45	35.62
**V156I**	19.07	4.2	4.5	Neutral	19.56	17.27
**S158C**	21.91	−0.8	2.5	Neutral	18.92	15.53
**V169I**	19.07	4.2	4.5	Neutral	3.50	23.15
**M180I**	2.33	1.9	4.5	Neutral	8.26	10.11
**R200K**	−2.77	−4.5	−3.90	Neutral	53.36	45.24
**V235A**	−36.71	4.2	1.8	Neutral	0.00	0.00
**V235I**	19.07	4.2	4.5	Neutral	0.00	0.00
**V248I**	19.07	4.2	4.5	Neutral	0.00	0.00
**Q258E**	−3.76	−3.5	−3.50	Neutral	28.50	33.14
**N262S**	21.30	−3.5	−3.50	Neutral	27.20	12.38
**S268T**	30.45	−0.8	−0.7	Neutral	29.04	26.75
**G273S**	48.09	−0.4	−0.8	Neutral	39.09	29.78
**T275I**	43.58	−0.7	4.5	Neutral	59.22	62.29
**D276E**	24.57	−3.50	−3.50	Neutral	32.07	40.53
**T279A**	−23.69	−0.7	1.8	Neutral	16.70	18.92

MUT, the position, and mutation in the amino acid one-letter code. %VOL, the percentual of volume change using the residue in the prototypic Faulkner strain. 1-Hydropathy and 2-Hydropathy, the hydropathy parameter using the Kyte and Doolittle scale [29] for the prototypic residue and the mutation, respectively. The Provean result, the Provean algorithm classification for the mutation. Faulkner SASA% and Mutation SASA%, the percentual change in the solvent-accessible surface area (SASA) of amino acid residue in the asymmetric unit using as reference the SASA value for the

maximum surface exposure. Residues with a SASA value of less than 10% are colored in red, with the high-frequency mutations highlighted in yellow. NC, not calculated, The Faulkner SASA% and Mutation SASA% values were not calculated for residues G4, A6, I7, and R9 due to the location at the disordered N-terminal, which is not present in the structural models.
